# An optimized purified inactivated Zika vaccine provides sustained immunogenicity and protection in cynomolgus macaques

**DOI:** 10.1038/s41541-020-0167-8

**Published:** 2020-03-12

**Authors:** Valérie Lecouturier, Vincent Pavot, Catherine Berry, Arnaud Donadieu, Aymeric de Montfort, Florence Boudet, Bachra Rokbi, Nicolas Jackson, Jon Heinrichs

**Affiliations:** 1 Research & Development, Sanofi Pasteur, Marcy l’Etoile, France; 2 Discovery Drive, Sanofi Pasteur, Swiftwater, PA USA; 3 Present Address: CEPI, London, UK

**Keywords:** Viral infection, Drug development

## Abstract

The recent spread of Zika virus (ZIKV) through the Americas and Caribbean and its devastating consequences for pregnant women and their babies have driven the search for a safe and efficacious ZIKV vaccine. Among the vaccine candidates, a first-generation ZIKV purified inactivated vaccine (ZPIV), adjuvanted with aluminum hydroxide, developed by the Walter Reed Army Institute of Research (WRAIR), has elicited high seroconversion rates in participants in three phase-I clinical trials. In collaboration with the WRAIR, Sanofi Pasteur (SP) optimized the production scale, culture and purification conditions, and increased the regulatory compliance, both of which are critical for clinical development and licensure of this vaccine. Using a clinical batch of the first-generation ZPIV as a benchmark, we report that different doses of the optimized vaccine (ZPIV-SP) elicited sustained neutralizing antibodies, specific T- and memory B-cells, and provided complete protection against a ZIKV challenge in cynomolgus macaques. These data provide evidence that the ZPIV-SP vaccine performs at least as well as the ZPIV vaccine, and provide support for continued development in the event of future ZIKV outbreaks.

## Introduction

After its discovery in Africa in 1947, Zika virus (ZIKV) was reported to be responsible for minor infections on the African continent and in South Asia^1^. In 2015, it spread to South America where it caused widespread disease, particularly in pregnant women, causing severe congenital abnormalities and death among fetuses and infants^2,3^. There have also been reports of Guillain-Barré syndrome and other neurologic disorders following ZIKV infection in adults^4,5^. ZIKV, like other members of the flavivirus genus to which it belongs, is transmitted by *Aedes* mosquitoes, but unlike the other flaviviruses, it exhibits tropism for cells of the neural lineage and can be transmitted sexually^6,7^.

Although there has been a strong decline in new ZIKV infections in South and Central America, their serious impact on fetuses and infants and the extended distribution of ZIKV’s transmission vector, *Aedes aegypti*, maintain Zika as a public health concern^8^. The threat of future outbreaks is considered as a real risk, based on recent ZIKV infections reported in Asia^9–11^. Consequently, many organizations have undertaken the development of Zika vaccine candidates, using both traditional and more novel vaccine platform technologies^12,13^.

Several ZIKV vaccine candidates have been shown to elicit protective antibodies in mice and non-human primates (NHP), including a ChimeriVax-Zika vaccine developed by Sanofi Pasteur^12,14^. Some candidate vaccines have also been assessed in humans, including DNA vaccines from Inovio and the National Institute of Allergy and Infectious Diseases (NIAID) Vaccine Research Center (VRC, NIH), and a purified inactivated ZIKV vaccine (ZPIV) developed by the Walter Reed Army Institute of Research (WRAIR)^15–17^. The ZPIV candidate contains a purified, formalin-inactivated ZIKV adsorbed onto an aluminum hydroxide (AlOOH) adjuvant. The vaccine-strain is derived from the PRVABC59 strain isolated in Puerto Rico in 2015 (ZIKV-PR), which is representative of the currently circulating Asian lineage^18^. Studies in mice and rhesus macaques demonstrated that ZPIV was immunogenic and protective from ZIKV challenge for at least 1 year^19–21^. The results from three phase-I studies in healthy adult volunteers demonstrated that the ZPIV candidate was well-tolerated and induced detectable neutralizing antibody titers in 92% of the individuals tested^16^.

However, as this vaccine does not meet quality standards required for large-scale deployment and is not produced in sufficient quantities for an epidemic, it is not suitable for further development. In collaboration with the WRAIR, Sanofi Pasteur (SP) has optimized the drug substance and drug product process of the first-generation ZPIV. This involved establishing a complete seed lot system and using a more stringent method of purification and inactivation in an entirely animal-product-free process. SP also developed the analytical tools to document the consistency of production. The immune responses of the resulting ZPIV-SP vaccine were then compared to those of the ZPIV vaccine.

The optimized vaccine candidate has been shown to induce robust seroneutralizing antibody responses and to provide protection against a homologous ZIKV challenge in immunocompetent BALB/c mice^22^. Compared with the first-generation ZPIV, ZPIV-SP also showed improved immunogenicity and efficacy in A129 mice, which are interferon-receptor deficient, and therefore more permissive to ZIKV infection. In addition, unlike the first-generation vaccine, ZPIV-SP did not induce any detectable IgG response in mice against the highly immunogenic non-structural protein 1 (NS1), indicating that most of this viral antigen was removed during the optimized purification process.

In this article, we report the assessment of the immunogenicity and efficacy of the ZPIV-SP vaccine in cynomolgus macaques that are phylogenetically much closer to humans than rodents and naturally susceptible to ZIKV infection^23,24^. We evaluated the immunogenicity of three different vaccine dose levels compared with that of the first-generation ZPIV candidate. We also assessed the immunogenicity of a booster dose at 6 months and protection from a homologous challenge with the wild-type (wt) ZIKV-PR strain.

## Results

### Clinical monitoring

**Fig. 1:**
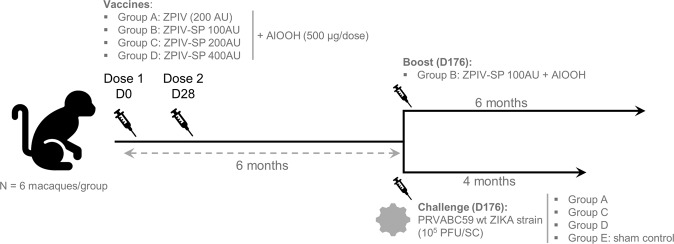
Schematic representation of the study schedule. Four groups of six cynomolgus macaques were immunized intramuscularly with the first-generation ZPIV (group A: 200 AU) or optimized ZPIV-SP (group B: 100 AU, group C: 200 AU or group D: 400 AU) on day 0 (D0) and on D28. Six months later (D176), the six macaques in group B received a booster dose. The other groups were used for Zika virus challenge.

Cynomolgus macaques were immunized with three-dose levels (100, 200, and 400 antigenic units, AU) of the optimized ZPIV-SP in comparison to 200 AU of the first-generation ZPIV previously tested in human clinical studies (Fig. 1). No local or systemic reactions were observed after any injection in any animal. Throughout the study, the macaques’ body weight either increased or remained stable.

### Humoral responses

**Fig. 2:**
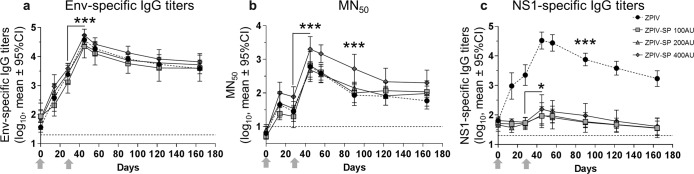
Humoral immune response to the ZPIV vaccines (mean log_10_ and 95% confidence intervals). **a** ZIKV Env-specific IgG ELISA titers, **b** ZIKV-specific microneutralization (MN_50_) titers, and **c** NS1-specific IgG ELISA titers following IM vaccination of six cynomolgus macaques with each dose: 100 AU, 200 AU, or 400 AU of ZPIV-SP or 200 AU of ZPIV on day 0 and day 28 (ANOVA *P-value < 0.05; ***P-value < 0.001). Dotted lines: limit of quantification. Arrows: vaccination on D0 and D28.

All vaccinated animals developed ZIKV envelope (Env)-specific binding antibodies, as measured by ELISA, and ZIKV-specific neutralizing antibodies, as quantified by microneutralization (MN_50_) assay. Env-specific IgG titers were not significantly different between groups and mean titers peaked at 4.5 log_10_ ELISA units (EU) 2 weeks post-dose 2 and then stabilized at D122 at 3.7 log_10_ EU (Fig. 2a).

The MN_50_ titers were significantly higher at all time-points in macaques immunized with the ZPIV-SP 400 AU dose (*P*-value < 0.001) with a threefold mean increase compared with the other dose levels of ZPIV-SP (Fig. 2b and Supplementary Fig. 1). ZPIV-SP 200 AU and ZPIV (5 µg/200 AU) induced similar neutralizing titers. In all groups, the MN_50_ titers significantly increased after dose 2 (*P*-value < 0.001) and peaked on D45 (mean titers: 3.3 log_10_ and 2.8 log_10_ for 400 AU and the other groups, respectively) and stabilized at D122 (mean titers: 2.3 log_10_ and 1.8 log_10_ for 400 AU and the other groups, respectively).

**Fig. 3:**
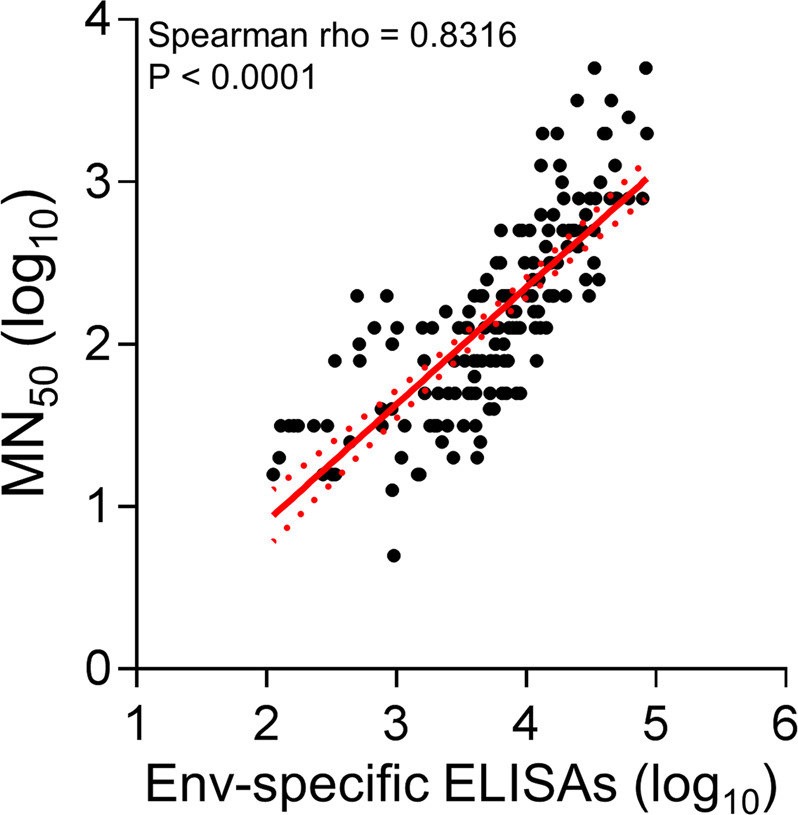
Correlation of MN_50_ and Env-specific IgG ELISA titers. Correlation was observed at all time points, in all vaccine groups. *P*-value for Spearman rank-correlation test. Line: linear regression; dotted lines: 95% confidence intervals.

Binding antibody titers correlated with neutralizing antibody titers in the ZPIV-vaccinated animals for all vaccine groups and at all time-points (Spearman rho = 0.8316; *P*-value < 0.0001; Fig. 3).

As most of the NS1 antigen was removed from the ZPIV-SP vaccine (Supplementary Tables 1 and 2) and no detectable anti-NS1 responses were previously observed in mice^22^, we sought to confirm the absence of those responses in the macaque model. The mean NS1-specific IgG titers for each group and at each time-point are shown in Fig. 2c. The NS1-specific IgG responses were low in macaques immunized with ZPIV-SP, irrespective of the dose, confirming previous mouse results. A slight “booster effect” was observed on D45 for ZPIV-SP at 200 AU and 400 AU (*P*-values < 0.05) but the titers remained low and returned to baseline levels on D90. In contrast, macaques immunized with ZPIV (containing about 100 ng of NS1/dose, Supplementary Table 1) developed high-NS1-specific IgG, which peaked on D45, and were still around 3 log_10_ on D164 (*P*-value < 0.001).

### Memory B-cell responses

**Fig. 4:**
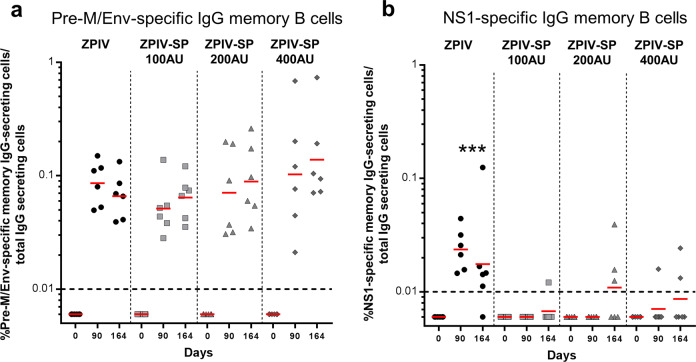
IgG memory B-cell ELISpot responses in PBMCs from ZPIV and ZPIV-SP-immunized macaques. **a** Env-specific memory B-cell ELISpot results, **b** NS1-specific memory B-cell ELISpot results at baseline, day 90 and day 164 following IM vaccination of cynomolgus macaques with 100, 200, or 400 AU of ZPIV-SP or 200 AU of ZPIV on day 0 and day 28. Bar = Geometric mean. (ANOVA ***P-value < 0.001). Dotted line = responder cutoff.

ZIKV-specific circulating memory B cells were quantified by ELISpot assay in peripheral blood mononuclear cells (PBMCs) from the immunized macaques on D90 and D164 after the first injection to assess the vaccine-induced long-term immune responses (reactive humoral memory). PrM/Env-specific IgG memory B cells were detected in all groups, at similar levels (geometric means ranging from: 0.05 to 0.1% of total IgG secreting cells) (Fig. 4a). ZPIV vaccine induced low but significant NS1-specific IgG memory B cells at both time points while such responses were detected only in a few animals in the ZPIV-SP vaccine groups (*P*-value < 0.001 (Fig. 4b)).

### T-cell immunity

Both CD4^+^ T_H_1 and T_H_2 cells exert essential helper functions and are critical for B-cell activation and differentiation^25^. Interferon (IFN)-γ and interleukin (IL)-5 were selected to analyze T_H_1 and T_H_2 immune responses, respectively.

**Fig. 5:**
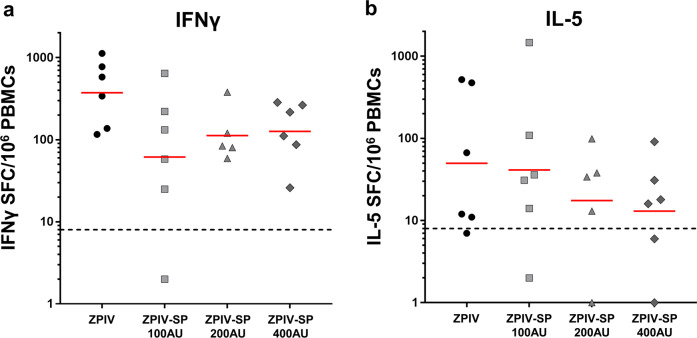
Characterization of cellular immune responses in macaques following vaccination. Env-specific T-cell ELISpot responses at D35 (7 days post-dose 2) in PBMCs from ZPIV and ZPIV-SP-immunized macaques. **a** IFNγ ELISpot results. **b** IL-5 ELISpot results. Bar represents Geometric mean. Dotted line represents responder cutoff.

Most vaccinated monkeys developed cellular immune responses, primarily to Env, as shown by the results from the IFNγ and IL-5 ELISpot assays 1 week after the second injection (Fig. 5). IFNγ spot-forming cells (SFC) ranged from 20 to 1000 per million PBMCs. A non-statistically significant trend for ZPIV to induce higher IFNγ responses to Env than ZPIV-SP vaccines was observed (*P*-value > 0.05). T-cell responses to capsid and prM were low to undetectable (Supplementary Fig. 2).

### Booster dose

**Fig. 6:**
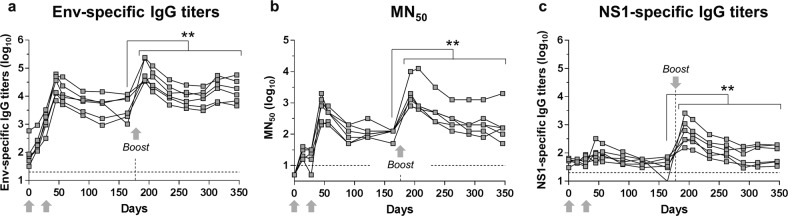
Humoral immune response after ZPIV-SP 100 AU booster injection. **a** ZIKV Env-specific IgG ELISA titers, **b** ZIKV-specific microneutralization (MN50) titers, and **c** NS1-specific IgG ELISA titers following IM vaccination of cynomolgus macaques with ZPIV-SP 100 AU on day 0, day 28, and a booster on day 176. ANOVA **P-value < 0.01. Horizontal dotted lines = limit of quantification. Arrows = vaccination on D0, D28, and boost. Vertical dotted line = boosting dose (D176).

A third dose was administered on D176 (5 months post-dose 2) to the ZPIV-SP 100 AU group to assess a potential booster effect. Fifteen days post-third dose, MN_50_ and Env-specific IgG titers were significantly boosted (ten-fold increase) compared with the pre-boost titers (*P*-value < 0.001) and remained two-fold higher until the end of the study at 6 months post-boost (*P*-value < 0.01) (Fig. 6). NS1-specific IgG responses were also significantly increased after the D176 boost (*P*-value < 0.01), indicating that even a low quantity of NS1 (<2.5 ng/dose, Supplementary Table 1) can induce a significant specific antibody response after three doses.

### Protective efficacy of ZPIV vaccines against wt-ZIKV challenge

We infected ZPIV-immunized (ZPIV, ZPIV-SP 200 AU and 400 AU groups) and sham control monkeys by the SC route with 10^5^ PFU of wt-ZIKV-PR (*n* = 6 macaques/group) to assess the vaccines protective efficacy against a ZIKV challenge. Viral loads after ZIKV challenge were quantitated by quantitative reverse transcription-polymerase chain reaction (qRT-PCR)^22^.

**Fig. 7:**
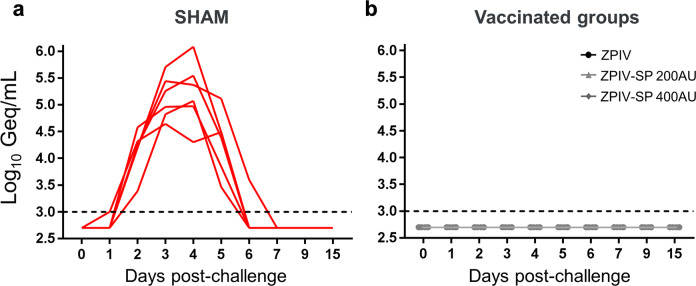
Protective efficacy of ZPIV vaccines in cynomolgus monkey. Viral loads were determined by the detection of ZIKV RNA in plasma from day 0 to day 15 post-Zika challenge. Protective efficacy of the ZPIV vaccines was shown by the detection of ZIKV RNA in the plasma of sham control macaques (**a**) but not in the plasma from the ZPIV-vaccinated macaques (ZPIV, ZPIV-SP 200 AU and 400 AU) (**b**). Dotted lines = limit of detection.

ZIKV RNA was detected in the plasma of all sham control macaques from D2 to D5/D6 post-challenge with a peak mean concentration of 5.2 log_10_ Geq/mL (titer range: 4.5 to 6.0 log_10_ Geq/mL; *n* = 6) on D3 and D4 (Fig. 7a). All 18 vaccinated monkeys were completely protected against ZIKV challenge, as shown by the lack of detection of viral RNA in the plasma samples (Fig. 7b). The lowest MN_50_ titer measured at the time of challenge was 1.4 log_10_ (group ZPIV-SP 200 AU).

Since ZIKV RNA was not detected in the saliva, ocular fluids or cerebrospinal fluid (CSF) from the sham control macaques, these analyses were not performed in the vaccinated macaques.

NS1 antigen was not detected in the plasma of any of the monkeys in the sham control group at peak viremia, suggesting that none or only low levels (< limit of detection) of NS1 antigen were present in the blood during viral replication.

The sham group displayed decreased body temperatures at D10 post-challenge (mean 35.2 °C) compared with D0 (mean 37 °C) while all protected vaccinated animals had body temperatures in the normal range (between 36 and 38 °C) (Supplementary Fig. 3)^26^.

### Immune response following wt-ZIKV challenge

**Fig. 8:**
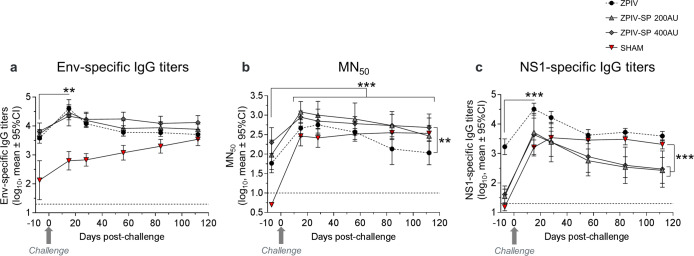
Follow-up of post-challenge humoral immune responses (log_10_ mean ± 95% confidence interval). **a** Env-specific IgG ELISA titers, **b** ZIKV-specific microneutralization (MN_50_) titers, and **c** NS1-specific IgG ELISA titers following challenge of cynomolgus macaques (*n* = 6/group) on day 176 by the SC route with 10^5^ PFU of PRVABC59 wt-ZIKA strain. (ANOVA **P-value < 0.01; ***P-value < 0.001). Dotted line = limit of quantification.

The wt-ZIKV challenge induced a transient increase in Env-specific IgG titers in all vaccinated groups (Fig. 8a). A durable increase in neutralizing responses in ZPIV-SP (200 and 400 AU) immunized monkeys was observed whereas only a transient increase was shown in the ZPIV group (Fig. 8b). In both ZPIV-SP groups, neutralizing titers on D112 (4 months post-challenge) were significantly higher than the pre-challenge titers (threefold increase, *P*-value < 0.001), unlike in the ZPIV group. MN_50_ titers were significantly higher in the ZPIV-SP 400 AU group compared with the ZPIV group (200 AU) at 3 months post-challenge (*P*-value < 0.01).

NS1-specific IgG responses increased in all groups after challenge (Fig. 8c, *P*-value < 0.001). In protected macaques (ZPIV and ZPIV-SP groups), NS1 antibodies decayed rapidly by tenfold, 1 month after the peak, whereas NS1-specific IgGs remained elevated until the end of the study in infected macaques (sham control group). NS1-specific IgGs were significantly lower in ZPIV-SP immunized groups than in the sham group from D56 (2 months post-challenge) to D112 (4 months post-challenge, end of the study) (*P*-value < 0.001).

**Fig. 9:**
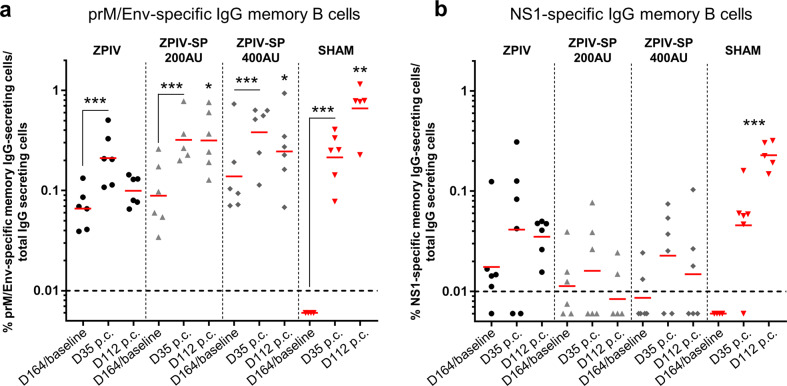
Post-challenge memory B-cell immune responses follow-up. **a** prM/Env-specific memory B-cell ELISpot responses, **b** NS1-specific memory B-cell ELISpot responses at baseline, day 35 and day 112 following ZIKV challenge in cynomolgus macaques with 10^5^ PFU of PRVABC59 wt-ZIKA strain. Bar = Geometric mean. (ANOVA *P-value < 0.05; **P-value < 0.01; ***P-value < 0.001). Dotted line = responder cutoff. p.c. = post-challenge.

On D35 post-challenge with wt-ZIKV, the number of prM/Env-specific IgG memory B cells increased in all groups compared with the pre-challenge levels on D164 (*P*-value < 0.001 (Fig. 9a)). In the ZPIV group, the number of prM/Env-specific memory B cells declined 4 months post-challenge (D112), but were stable for both the ZPIV-SP 200 AU and 400 AU groups. On D112, there were significantly more prM/Env-specific memory B cells in the ZPIV-SP 200 and 400 AU groups than in the ZPIV group (*P*-values < 0.05). In the sham control group, at D112 the number of prM/Env-specific memory B cells was higher than in the vaccinated groups (*P*-value < 0.01 (Fig. 9a)).

The NS1-specific memory B-cell responses transiently increased post-challenge in some macaques in the vaccinated groups (D35 post-challenge) and were maintained at high levels at 4-months post-challenge in the sham group (*P*-value < 0.001 (Fig. 9b)).

**Fig. 10:**
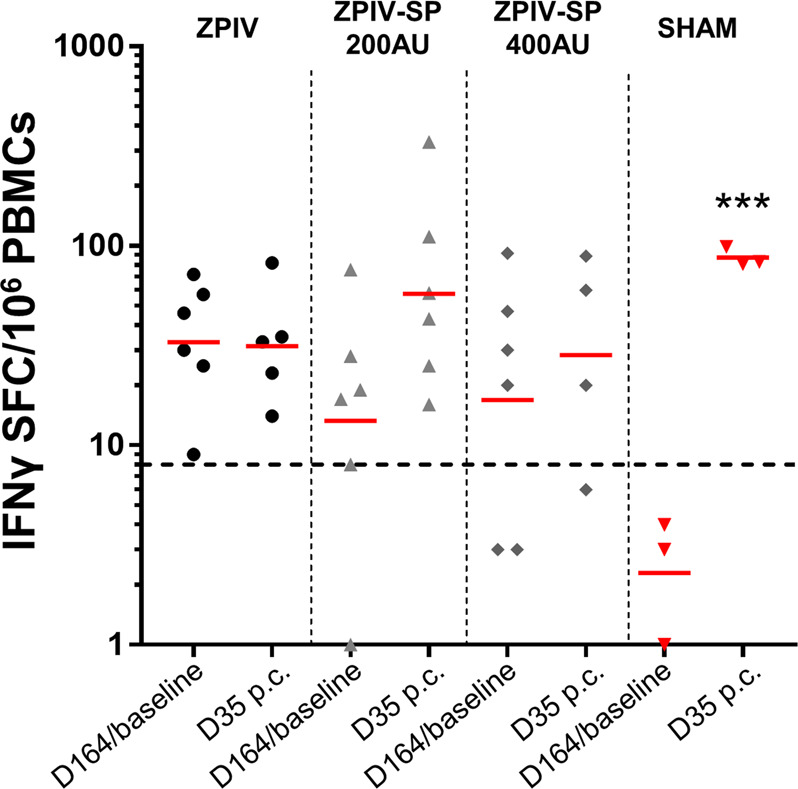
Characterization of cellular immune responses in macaques following challenge. Env-specific IFNγ ELISpot responses before and 35 days post ZIKV challenge. Bar = Geometric mean. (ANOVA ***P-value < 0.001). Dotted line = responder cutoff. p.c. = post-challenge.

Analyses of the T-cell-mediated immunity by ELISpot assay showed that most vaccinated monkeys still had detectable Env-specific IFNγ-secreting cells 5-months post-dose 2 (D164 = 12 days before challenge), and that these responses were not significantly boosted by the ZIKV-PR challenge (Fig. 10). There was a significant induction of Env- and capsid-specific IFNγ responses in the sham control group following challenge (*P*-value < 0.001) (Fig. 10 and Supplementary Fig. 4).

## Discussion

Our results demonstrate that two injections of the optimized ZPIV-SP adjuvanted with AlOOH provided complete protection against ZIKV-PR challenge in 100% (*n* = 12) of cynomolgus macaques 6 months after immunization, as did the first-generation ZPIV. No specific adverse effects related to the vaccines were reported based on local and systemic observations. Seroconversion was shown in all macaques and Env-specific IgG and neutralizing responses remained detectable at 6 months.

The higher dose of ZPIV-SP (400 AU) induced higher neutralizing antibody responses compared to ZPIV-SP 200 AU, which had a similar profile to the ZPIV benchmark vaccine (5 µg/200 AU). The antibody profiles of the ZPIV and ZPIV-SP vaccines in cynomolgus macaques confirm those previously reported in rhesus macaques^20^. This is in contrast to observations in the mouse models in which ZPIV-SP was more immunogenic than ZPIV at equivalent doses^22^.

We also demonstrated robust memory B-cell responses elicited by both the first-generation ZPIV and the ZPIV-SP vaccines, which may be crucial for long-term protection via activation of circulating memory B cells during ZIKV infection (reactive humoral memory)^27^.

Protective adaptive immunity to ZIKV has been mainly attributed to neutralizing antibodies but the role of cytotoxic CD8^+^ T cells, as well as CD4^+^ T cells and IFNγ signaling in antibody-mediated resistance to ZIKV infection has also been suggested^28,29^. The majority of the vaccinated macaques developed T-cell immune responses, primarily to Env, as measured by IFNγ and IL-5 ELISpot assays, similar to the results reported in rhesus macaques^19^.

In addition, our results show that a third dose of ZPIV-SP 100 AU, given 6 months after the first dose, elicited a robust booster effect on humoral responses, with a significant increase in Env-specific IgG and neutralizing antibody titers. Four months after this third dose, the MN_50_ titers stabilized and were still twofold higher than that achieved after the second dose. These data suggest that a three-dose immunization schedule at 0, 1, and 6 months with ZPIV-SP, at a dose level as low as 100 AU, could, not only maintain, but also increase the neutralizing antibody response, potentially contributing to a longer duration of protection.

Consistent with previous data from mouse studies, ZPIV-SP induced low levels of NS1 antibodies, detected only after the third dose, confirming the low NS1 content in the optimized vaccine^22^.

The cynomolgus macaques vaccinated twice with ZPIV or ZPIV-SP (200 or 400 AU) were fully protected against a homologous challenge with the wt-ZIKV-PR strain administered 5 months after the second vaccine dose. This protection was demonstrated by the absence of detectable viral RNA in plasma samples and stable body temperatures in the vaccinated, challenged animals compared with the high RNA levels and decrease in body temperature in the sham control group.

In contrast to what has been reported in rhesus macaques, and in cynomolgus macaques, we did not detect viral RNA in saliva, ocular fluids or CSF from the sham control macaques after challenge, suggesting that the dissemination of ZIKV is not systematic in cynomolgus macaques, unlike what has been reported in rhesus macaques^24,30,31^. Both the route of infection used (intranasal or intragastric) and the viral strain used in the previous study in cynomolgus macaques showing viral dissemination in tears, saliva and urine differed from our study, which may explain the differences seen in viral dissemination^24,31^.

We assessed the NS1-specific post-challenge antibody response as a biomarker of infection and observed a transient increase of anti-NS1 titers in all vaccinated macaques, compared with higher and durable titers in sham macaques. This transient increase could be explained by a low level of viral replication (not detected with qRT-PCR in plasma) or the presence of NS1 antigen in the viral challenge dose. We confirmed the presence of 10 ng of NS1 antigen in the wt-ZIKA challenge dose explaining the anti-NS1 IgG response in vaccinated, protected animals but we cannot exclude a limited viral replication.

Our results in an NHP model confirm those previously reported in mice, namely that the optimized ZPIV-SP vaccine is at least as immunoprotective as the first-generation ZPIV that has previously been tested in clinical trials^16,22^. These observations suggest that the purification process used to prepare ZPIV-SP, which included removal of NS1, did not have a negative impact on vaccine efficacy. Moreover, the absence of NS1 in the vaccine might represent an advantage during clinical development, where an NS1-specific serological assay could be used to distinguish between vaccination and natural infection^32,33^.

Development of an anamnestic antibody response was also observed after ZIKV challenge in the immunized groups, as shown by the increase in Env-specific IgG and neutralizing antibody titers, indicating that the inactivated vaccines were able to prime the immune system of the macaques successfully. Interestingly, after the challenge, the increase in MN_50_ titers and Env-specific memory B cells remained more stable in the macaques vaccinated with ZPIV-SP than in those vaccinated with ZPIV. The titers achieved in the challenged-vaccinated animals were in the same range as those observed in the boosted, unchallenged monkeys shortly after the booster dose. These findings suggest that natural exposure to ZIKV may boost the vaccine-induced response in ZIKV-endemic countries.

No protective threshold for MN_50_ titers could be established in our study since all immunized monkeys were protected against viral challenge. However, the results suggest that the lowest MN_50_ titer of 1.4 log_10_ measured before challenge was sufficient to confer protection against viremia. This is consistent with the low protective antibody levels previously reported for other flaviviruses such as Japanese encephalitis virus and yellow fever virus^34^. The lowest protective titer found in our study is slightly lower than the 2.0 log_10_ threshold previously reported in rhesus macaques for ZPIV, DNA, and adeno recombinant ZIKV vaccine candidates and the 3 log_10_ EC_50_ reported to confer 70% protection after ZIKV DNA immunization^20,35^. The difference could be due to the animal model (cynomolgus vs. rhesus monkeys), the vaccine platform (ZPIV vs. DNA) or the assay (MN_50_ vs. EC_50_) used. Also, we cannot exclude the contribution of other immune effectors that could promote viral control such as virus-specific CD4/CD8 T-cells as shown for ZIKV^29^. In fact, Env-specific IFNγ T-cell responses induced shortly after immunization with all ZPIV vaccines, were still detectable prior to challenge and may have contributed to protection.

Taken together, these results demonstrate durable and robust protection against wt-ZIKV challenge by an optimized inactivated ZIKV vaccine adjuvanted with AlOOH in cynomolgus macaques. These results in macaques confirm the prior results in mice and extend them to an NHP model using vaccine doses and an administration route applicable to humans.

The first-generation adjuvanted ZPIV, developed in response to an urgent situation, is currently being evaluated in phase-I clinical trials^36^. The new drug substance and drug product processes developed up to pilot scale are quality compliant and deliver a high quality and optimized ZPIV-SP vaccine candidate. Combined with its excellent performance in animal models this indicates that the vaccine would be appropriate for use for accelerated clinical development in the event of future ZIKV outbreaks.

## Methods

### ZPIV and ZPIV-SP vaccine formulations

The ZPIV candidate (phase-I clinical batch) was provided by WRAIR^16^. The vaccine was supplied in a liquid form, ready for injection, at 5 µg of proteins per dose (500 µL), corresponding to 200 antigenic units (AU), formulated with AlOOH. The optimized vaccine (ZPIV-SP) was prepared at Sanofi Pasteur (Marcy l’Etoile, France) using the WRAIR process as a starting point with the following improvements. Briefly, after initial amplification of ZIKV-PR in Sanofi Pasteur Vero cells, viral RNA was extracted and transfected into Sanofi Pasteur’s serum-free (SPSF) Vero cells. Recovered virus was amplified, plaque-purified twice and further amplified to generate a pre-Master Seed Lot from which a Master and a Working Seed Lot were derived. The virus was produced in a 180 L bioreactor using SPSF Vero cells. The virus was then clarified, purified by ultracentrifugation with a modified cutoff and chromatography, and inactivated by formalin treatment. The parameters of the purification and inactivation steps were optimized compared with the conditions used by WRAIR. The drug product was adjusted for ZIKV Envelope (E) antigenic content by ELISA to 400 AU/mL (corresponding to 10 µg/mL of ZPIV proteins) and lyophilized. The lyophilized vaccine was adjusted to 100, 200 or 400 AU per dose (500 µL) and resuspended in an AlOOH gel (500 µg/dose—Brenntag Biosector, Denmark). AU was defined as the envelope (Env) antigenic content measured by ELISA.

### Vaccination schedule

Four groups of six flavivirus-negative male cynomolgus macaques (*Macaca fascicularis* - Noveprim), aged 2 years, received 500 µL of first-generation ZPIV (group A: 200 AU) or optimized ZPIV-SP (group B: 100 AU, group C: 200 AU or group D: 400 AU) intramuscularly (IM) in the right quadriceps on day 0 (D0) and in the left quadriceps on D28. Six months later (D176), the six macaques in group B that had received 100 AU of ZPIV-SP, received a booster dose of the same formulation and were followed for 6 months (Fig. 1). The other groups were used for the viral challenge.

### Clinical monitoring

Immediate reactions were observed within 30 min and potential local reactions at the injection site were observed over the 7 days following each immunization. The macaques were monitored every day and symptoms such as decreased food intake, restricted mobility, polypnea, local and systemic reactions were recorded throughout the study. Body weight and body temperature (using transponder chips) were recorded at baseline and at regular intervals throughout the study.

### Blood sampling post-immunization for immunogenicity assessment

Sera were collected at different time points over 6 months post-dose 1 and 2 (D0 to D164) and post-boost for group B (D15 to D170) to assess humoral responses, including ZIKV neutralizing antibodies by a MN_50_ assay and ZIKV Env and NS1-specific IgG by ELISA.

PBMCs were collected at baseline, D7, D35, D90, and D164 to assess cellular-mediated immunity and memory B-cell responses.

### Challenge with wild-type ZIKV-PR

Five months post-dose 2 (D176), the macaques in groups A, C, D, and six naive macaques (group E: sham control) were challenged with 10^5^ plaque forming units (PFU diluted in phosphate-buffered saline (PBS)) of wt-ZIKV-PR (strain PRVABC59 from CDC) by subcutaneous (SC) injection in the deltoid region of the right arm and under anesthesia (Zoletil®) (Fig. 1). The challenge dose and route were selected based on results from a previous study (unpublished results).

### Biological samplings post-challenge

Plasma was collected every day for 7 days and on D9 and D15 post-challenge for virologic assays. CSF (100–300 µL) was collected from the *cisterna magna* on D7, D15 and D28 post-challenge. Absence of blood contamination in CSF was confirmed through visual inspection and samples were stored at –80 °C. Saliva was collected every day for 15 days and ocular fluids were collected with a swab on D7 and D15 post-challenge. The swabs were placed into tubes containing 500 μL of RNA Later (Invitrogen, USA) and were mixed by vortexing. The swabs were then discarded, and eluates were stored at –80 °C until analyzed.

For immunologic assays plasma was collected at multiple time points from D15 to D112 and PBMCs were collected on D35 and D112.

### Viremia and viral loads in biological fluids post-challenge

ZIKV RNA in plasma, saliva, ocular fluid and CSF samples from groups A, C, D, and E was quantified post-challenge using a qRT-PCR targeting the NS5 gene^22^. Total genomic RNA was first extracted from samples with a Macherey Nagel NucleoSpin® 96 virus kit (Macherey Nagel, Germany) on a Tecan Evoware automated RNA-extraction workstation according to the manufacturer’s instructions and eluted in nuclease-free water.

### Sandwich ELISA to quantify ZIKV NS1 antigen

Residual ZIKV NS1 antigen content was quantified by a sandwich ELISA in the virus preparation used for challenge. It was also measured in undiluted and diluted (1:30) plasma samples collected at baseline, D1, D2, D3, D4, and D5 from all challenged groups as an indirect method to assess viral replication.

Briefly, 96-well ELISA plates were coated with a mouse monoclonal antibody specific for the ZIKV NS1 protein (clone B-J5 R&D Biotech, France) in phosphate-buffered saline 1 × (PBS). Unbound sites were then blocked for 1 h at 37 °C with PBS-Tween20 in 1% milk (PBS-Tw-M). The plates were washed with PBS-Tween20 between incubation steps. Test samples were serially diluted twofold in PBS-Tw-M and incubated in the wells for 1 h at 37 °C. A second mouse monoclonal antibody horseradish peroxidase (HRP)-conjugate directed to ZIKV NS1 protein (clone B-M6 R&D Biotech, France) was added and incubated for 90 min at 37 °C. Then, the plates were incubated in the dark for 45 min at room temperature (RT) with a ready-to-use TetraMethylBenzidine (Tebu-Bio Laboratories, France) substrate. Reactions were stopped with 1 N HCl (VWR Prolabo). The optical density (OD) was measured at 450–650 nm with an automatic plate reader. A ZIKV NS1 recombinant protein (Native Antigen, UK) was used to establish a standard curve to determine the NS1 content of the test samples. The protein concentration was determined as the average of all individual concentrations for the OD value range of 0.2 to 3.0. The limit of detection (LOD) of the assay was 5 ng/mL.

### Immunologic assays

#### Envelope- and NS1-specific IgGs

Envelope- and NS1-specific IgGs were assessed by ELISA in 96-well plates coated with recombinant E protein (Meridian Life Science Inc., Memphis, USA) or recombinant non-structural protein 1 (NS1) protein (Native Antigen Company, Oxford, UK) in carbonate buffer, pH 9.6. Following blocking with PBS-Tween20-milk for 60 min at 37 °C, twofold diluted serum samples were added and incubated for 90 min at 37 °C. Washing steps were performed between incubation steps with PBS-Tween. A goat anti-monkey IgG HRP-conjugate (Ref AAI42P, BIO-RAD, France) diluted in PBS-Tw-M at 1:5000 was added and incubated for another 90 min at 37 °C before color development with tetramethylbenzidine substrate (Tebu-Bio Laboratories, Le-Perray-en-Yvelines, France). Optical density was measured at 450–650 nm with an automatic plate reader. Titers for E-specific IgGs were calculated using an anti-ZIKV monkey reference serum regression curve. Titers for NS1-specific IgGs were calculated as the reciprocal dilution of the serum giving an optical density of 1 using the tendency function. The titer of the reference was previously calculated as the average of several determinations of the reciprocal dilution giving an optical density of 1.0. All titers were expressed in log_10_ ELISA Units (EU). The LOD was set at 1.3 log_10_ EU and an arbitrary titer of 1.0 log_10_ was assigned to each titer below the LOD.

#### ZIKV neutralizing antibodies

A high-throughput ZIKV MN_50_ assay was used for measuring ZIKV-specific neutralizing antibodies^22^. Briefly, heat-inactivated sera were serially diluted, mixed with ZIKV-PR and incubated at 37 °C for 75 min. The mixtures were then transferred to 96-well plates containing confluent Vero cell monolayers. Following incubation for 5 days, infected cells were stained with biotinylated pan-flavivirus 4G2 mAb (HB112, Biotem, France) and visualized with 5-bromo-4-chloro-3-indolylphosphate/nitro blue tetrazolium in Levamisole substrate. Positive wells were defined as at least one colored infectious focus detected. For each dilution, the total number of negative wells was recorded and the reciprocal dilution corresponding to 50% of viral neutralization was calculated using the least square method and expressed as the neutralization log_10_ MN_50_ titer.

#### Memory B-cell enzyme-linked immunospot (ELISpot) assay

Cryo-preserved PBMCs were quickly thawed in a 37 °C water bath. A mixture of fetal calf serum (FCS) / DNAse (100 μg/mL) was slowly added to the PBMCs, before being transferred to RPMIc (RPMI/10% FCS/glutamine/antibiotic cocktail). After 1 h at 37 °C, PBMCs were counted using the ViaCount Guava kit according to the manufacturer’s instructions. The PBMCs were resuspended at 1 × 10^6^ cells/mL in polyclonal stimulation medium containing RPMIc with R848 (resiquimod) at 1 μg/mL and IL-2 at 10 ng/mL (both from Mabtech) and incubated at 37 °C with 5% CO_2_ for 4 days to allow differentiation of memory B cells into antibody-secreting cells (ASC)^37^.

Sterile 96-well Multiscreen-IP plates with PVDF membranes (Millipore) were pre-incubated with 35 µL of 35% ethanol, then washed three times with sterile PBS 1X before being coated with 100 μL of an anti-IgG human antibody (Mabtech clone 3850–3–1000) to detect total IgG secreting cells or pre-membrane and envelope (prM/Env) virus-like particles (VLP) ZIKV or NS1 ZIKV (The Native Antigen Company, UK). The plates were incubated overnight at 4 °C and then washed three times with sterile 1x PBS and saturated with RPMIc medium for 2 h at 37 °C. RPMIc medium was then removed and PBMCs were added at 2500 and 5000 cells in the antibody-coated capture wells, and at 200,000 and 400,000 cells in the ZIKV prM/Env VLP or ZIKV NS1-coated wells, in RPMIc medium. Each condition was tested in triplicate and plates were incubated for 20 h at 37 °C with 5% CO_2_.

The plates were washed twice in PBS/Tween 20 (0.05%) and three times with PBS and incubated with a biotinylated anti-human IgG Ab (Mabtech clone MT78/145-Biotin, Sweden) at 1 μg/mL in PBS 1 × /0.5% BSA at RT for 1.5 h. The plates were further washed five times in PBS, and phycoerythrin-labeled streptavidin (Sigma, France) diluted 1:100 with 0.5% PBS/BSA was added. The plates were incubated for 1 h at RT, then washed six times with PBS, dried and kept in the dark. The plates were read with the HLW20 reader (Microvision Instrument) using IRIS software and analyzed with Cosmic software. The results are expressed as the percentage of antigen-specific ASCs among total IgG ASCs.

#### T-cell ELISpot assays

ZIKV-specific T-cell immune responses were assessed by IFNγ and IL-5 ELISpot assays using pools of 15-amino-acid (aa) peptides with 11 aa overlaps covering the Env, prM and Capsid ZIKV proteins (JPT, Germany). Briefly, 96-well Multiscreen-IP plates with PVDF membranes (Millipore) were pre-treated with 35% ethanol, then coated overnight at 4 °C with 100 µL/well of anti-monkey/human IFNγ (Mabtech clone MT126L) or anti-human IL-5 (Mabtech clone TRFK5) at 10 µg/mL in sterile PBS 1×. After blocking with RPMIc (RPMI/10% FCS/Glutamine/antibiotic cocktail), 3 × 10^5^ thawed PBMCs were incubated in triplicate with 2 µg/mL of each ZIKV peptides or an irrelevant peptide pool (negative control) in the presence of anti-CD28 (mAb CD28.2) and anti-CD49d (mAb 9F10) from Biolegend as co-stimulators. PHA (Remel)/PMA (SIGMA) stimulation was used as positive control. Following an 18-h incubation at 37 °C, the plates were washed in PBS-BSA 0.5% and incubated with biotinylated anti-human IFNγ (Mabtech clone 7-B6-1) or biotinylated anti-human IL-5 (Mabtech clone 5A10) at 1 µg/mL, under 100 µL/well, for 2 h at RT, in the dark. After washing, plates were subsequently incubated with streptavidin-PE (Southern Biotech) for 1 h at RT, in the dark followed by six washes in PBS-BSA 0.5%.

The plates were read with the HLW20 reader (Microvision Instrument) using IRIS software and analyzed with Cosmic software. Results were expressed as the number of IFNγ or IL-5 spot-forming cells (SFC) per 10^6^ PBMCs.

### Statistical methods

All data were log-transformed prior to statistical analyses. From D0 to D164, the analyses of the humoral responses were performed using a longitudinal model of analysis of variances with repeated measurements at different time-points for each monkey. The time effect was modeled using a quadratic effect. To model the challenge and boost effects, a longitudinal model of analysis of variance was used for each read-out. For each monkey, repeated measurements at different time-points were taken into account in the model. Tukey or Dunnett adjustments were performed for multiple comparisons.

For the cell-mediated immunity, comparisons between groups or time-points were made by a one-way ANOVA or using a longitudinal model depending on the biological question.

All analyses were performed using SAS® v9.4 software at an alpha level of 0.05. *P*-values lower than this value indicated statistically significant differences.

### Ethical considerations

All animal experiments were performed in accordance with the Association for Assessment and Accreditation of Laboratory Animal Care accredited animal facilities, in compliance with the European Directive 2010/63 and French national regulations. The protocols were approved by the Sanofi Pasteur Ethical Committee for Animal Experimentation.

The macaques that were used in the challenge study were humanely euthanized at the end of the study (4 months post-challenge) for biosafety reasons, as ZIKV has been reported to be occasionally persistent in macaques^30^. Animals used for the booster study were reused in other research experiments.

### Reporting summary

Further information on research design is available in the Nature Research Reporting Summary linked to this article.

## Supplementary information

Supplementary Information

Reporting Summary

## Data Availability

The authors declare that the data supporting the findings of this study are available within the paper and its supplementary information files.
