# Supplementary material for: An optimized purified inactivated Zika vaccine provides sustained immunogenicity and protection in cynomolgus macaques
Source: NPJ Vaccines. 2020 Mar 12;5:19. doi: 10.1038/s41541-020-0167-8 (PMC7067768; doi:10.1038/s41541-020-0167-8)
Supplement: Supplementary file 1 — Supplementary Information [file 41541_2020_167_MOESM1_ESM.pdf]

Supplementary Information

An optimized purified inactivated Zika vaccine provides sustained immunogenicity and protection in cynomolgus macaques

Valérie Lecouturier[1]*, Vincent Pavot[1]*, Catherine Berry[1], Arnaud Donadieu[1], Aymeric de Montfort[1], Florence Boudet[1], Bachra Rokbi[1], Nicolas Jackson[1#] Jon Heinrichs[2]

*These authors contributed equally to this work

[1] Research & Development, Sanofi Pasteur, Marcy l'Etoile, France.

[2] Discovery Drive, Sanofi Pasteur, Swiftwater, PA, USA.

[#] Present address: CEPI, London, UK.

Corresponding author: Valérie Lecouturier, Research & Development, Sanofi Pasteur, 1541 Avenue Marcel Mérieux, 69280 Marcy l'Etoile, France (email: valerie.lecouturier@sanofi.com)

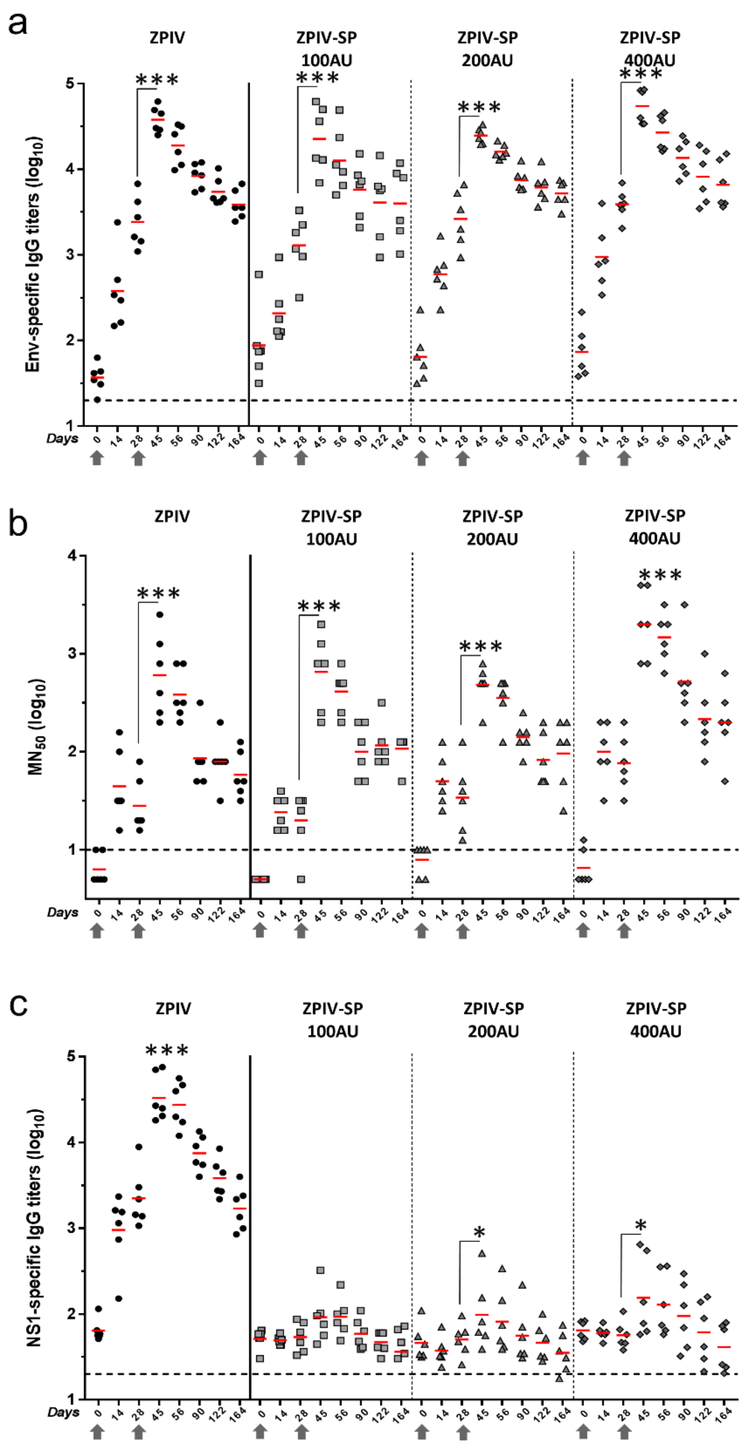

Supplementary Figure 1. Scatter plots of humoral immune response to ZPIV vaccines. (a) ZIKV Env-specific IgG ELISA titers, (b) ZIKV-specific microneutralization (MN_{50}) titers and (c) NS1-specific IgG ELISA titers following IM vaccination of cynomolgus macaques with 100 AU, 200 AU or 400 AU of ZPIV-SP or 200 AU of ZPIV on day 0 and day 28 (ANOVA **P*-value < 0.05; ****P*-value < 0.001). Bars = arithmetic mean. Dotted line = limit of quantification. Arrows = vaccination on D0 and D28.

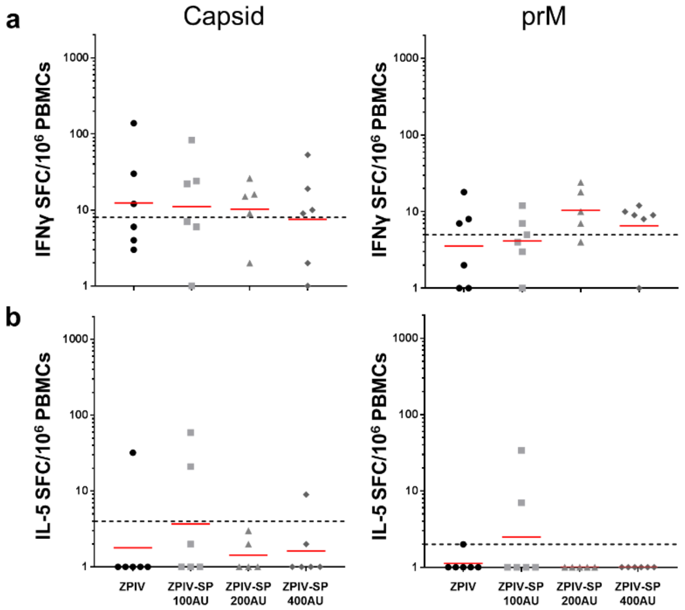

Supplementary Figure 2. Capsid and prM-specific T-cell ELISpot responses at D35 (7 days post-dose 2) in PBMCs from ZPIV and ZPIV-SP-immunized macaques. (a) IFNγ ELISpots. (b) IL-5 ELISpots. Bar = Geometric mean. Dotted line = responder cut-off.

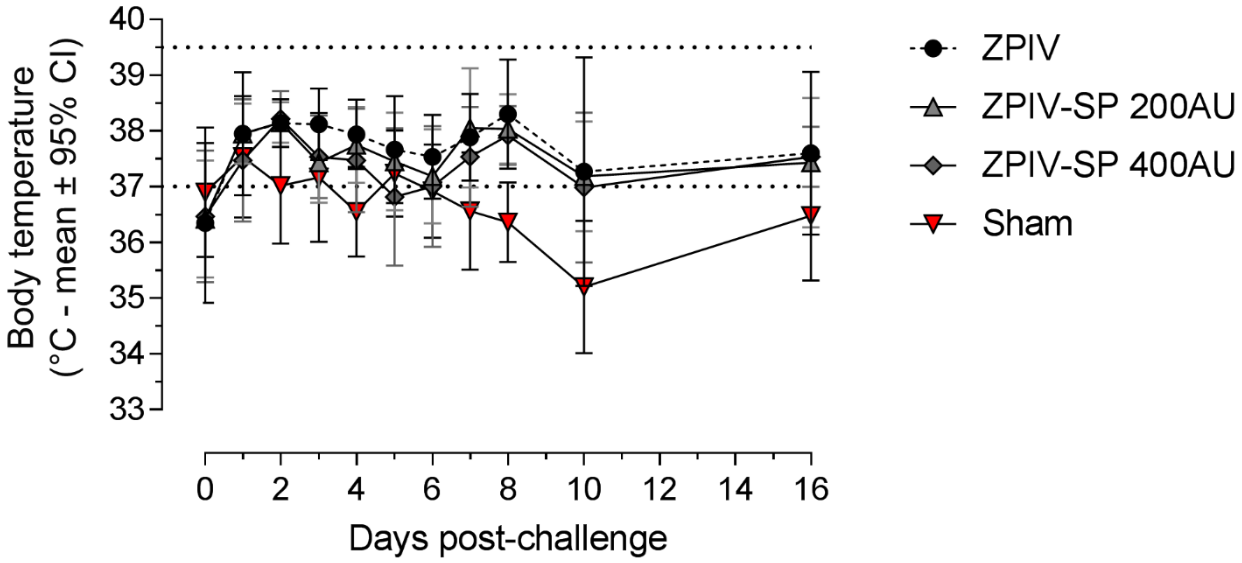

Supplementary Figure 3. Body temperature post-challenge. Dotted lines represent the normal body temperature range in cynomolgus macaques (n = 6/group).

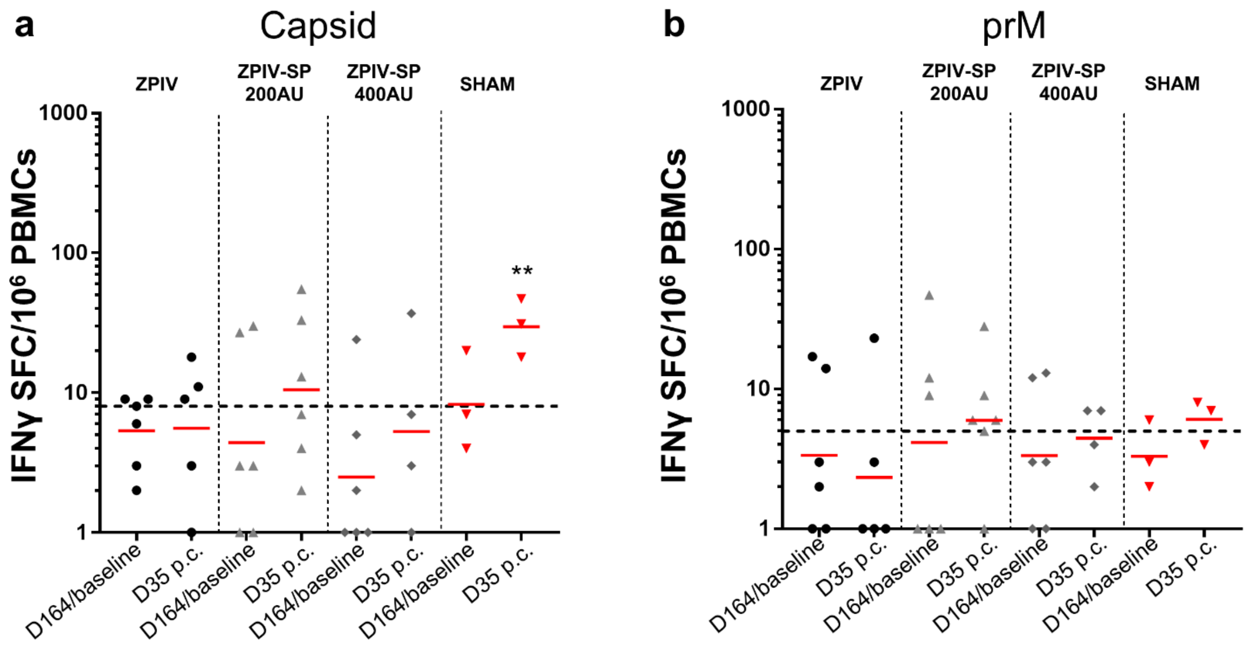

Supplementary Figure 4. Capsid and prM-specific IFNγ ELISpot responses before and after challenge. Bar = Geometric mean. Dotted line = responder cut-off. p.c. = post-challenge

Supplementary Tables

Supplementary Table 1. NS1 residual content in ZPIV and ZPIV-SP doses

Vaccine	Final NS1 content (ng/dose)
ZPIV	100
ZPIV-SP (200 AU)	ND (< 2.5ng)

ND: below level of detection

Supplementary Table 2. NS1 concentration (ng/mL) in successive ZPIV-SP production stages

ND: below level of detection

Crude harvest (ng/mL)	Clarified harvest (ng/mL)	Purified bulk (ng/mL)	Purified Concentrated bulk (ng/mL)
4300	380	ND (< 10)	10
